# Growth Characteristics of an Estuarine Heterocystous Cyanobacterium

**DOI:** 10.3389/fmicb.2017.01132

**Published:** 2017-06-16

**Authors:** Pablo Guimarães, João S. Yunes, Mariana Silvia Cretoiu, Lucas J. Stal

**Affiliations:** ^1^Department of Marine Microbiology and Biogeochemistry, Royal Netherlands Institute for Sea Research and Utrecht UniversityDen Burg, Netherlands; ^2^Programa de Pós-Graduação em Oceanografia Física, Química e Geológica, Universidade Federal do Rio Grande – FURGRio Grande, Brazil; ^3^Department of Aquatic Microbiology, University of AmsterdamAmsterdam, Netherlands

**Keywords:** akinete, benthic cyanobacterium, *Cylindrospermopsis raciborskii*, estuarine cyanobacterium, heterocyst, nitrogen fixation, salt tolerance

## Abstract

A new estuarine filamentous heterocystous cyanobacterium was isolated from intertidal sediment of the Lagoa dos Patos estuary (Brazil). The isolate may represent a new genus related to *Cylindrospermopsis*. While the latter is planktonic, contains gas vesicles, and is toxic, the newly isolated strain is benthic and does not contain gas vesicles. It is not known whether the new strain is toxic. It grows equally well in freshwater, brackish and full salinity growth media, in the absence of inorganic or organic combined nitrogen, with a growth rate 0.6 d^-1^. Nitrogenase, the enzyme complex responsible for fixing dinitrogen, was most active during the initial growth phase and its activity was not different between the different salinities tested (freshwater, brackish, and full salinity seawater). Salinity shock also did not affect nitrogenase activity. The frequency of heterocysts was high, coinciding with high nitrogenase activity during the initial growth phase, but decreased subsequently. However, the frequency of heterocysts decreased considerably more at higher salinity, while no change in nitrogenase activity occurred, indicating a higher efficiency of dinitrogen fixation. Akinete frequency was low in the initial growth phase and higher in the late growth phase. Akinete frequency was much lower at high salinity, which might indicate better growth conditions or that akinete differentiation was under the same control as heterocyst differentiation. These trends have hitherto not been reported for heterocystous cyanobacteria but they seem to be well fitted for an estuarine life style.

## Introduction

Intertidal sediments are located between the high- and low water marks and confine the area that is alternatingly immersed by seawater and exposed to the air. Depending on the location as well as the actual tidal range, wind direction and -force, the duration of immersion and exposure varies. This results in a plethora of environmental conditions. Because water potential and salinity may vary drastically due to desiccation and rain, while also temperature may fluctuate strongly, intertidal sediments are considered to be extreme environments. In addition, sandy intertidal sediments are usually low in nutrients. These intertidal areas often are the habitat of microbial mats that develop on the sediment surface worldwide (Stal et al., 1985). Intertidal coastal microbial mats are built by cyanobacteria, oxygenic phototrophic bacteria that use sunlight and water, and fix CO_2_ into organic matter, which forms the basis of a microbial foodweb (Stal, 2001). Many cyanobacteria are capable of fixing atmospheric dinitrogen (N_2_) and they have therefore access to an unlimited source of nitrogen that fertilizes the microbial community (Zehr, 2011).

Nitrogenase, the enzyme complex responsible for the reduction of N_2_ to 2NH_3_ (dinitrogen fixation), is in demand of a high amount of energy (16 ATP per N_2_ fixed) and low-potential reducing equivalents (8 reduced ferredoxin, FdH) and is sensitive to O_2_ and therefore functions only under near anoxic conditions (Fay, 1992). Only bacteria and a few archaea that possess the genetic capacity of synthesizing nitrogenase are capable of fixing dinitrogen. Dinitrogen fixation by eukarya has only been observed in symbiosis with bacteria (Kneip et al., 2007). Cyanobacteria satisfy the demand for energy (light) and reducing equivalents (H_2_O) to fix dinitrogen. However, the oxygen-evolving nature of cyanobacteria seems to be in contradiction with the oxygen sensitivity of nitrogenase and not compatible with it (Fay, 1992). Diazotrophic cyanobacteria have evolved various strategies to avoid inactivation of nitrogenase by oxygen (Gallon, 1992; Berman-Frank et al., 2003).

A unique strategy is the differentiation of cells that have lost the oxygenic photosystem 2 and possess a thick cell wall that limits the diffusion of gas (i.e., oxygen) (Muro-Pastor and Hess, 2012). These cells are called heterocysts that cannot differentiate back to vegetative cells and do not divide. Respiration in these heterocysts has a high affinity for oxygen, which keeps the O_2_ concentration close to zero and thereby providing the conditions for nitrogenase activity. Heterocystous cyanobacteria are filamentous and approximately 5% of the cells differentiate into heterocysts that are usually arranged at regular distances along the trichome providing the neighboring vegetative cells with fixed nitrogen. This strategy is also known as a spatial separation of oxygenic photosynthesis (in the vegetative cells) and dinitrogen fixation (in the heterocysts) (Fay, 1992). Some heterocystous cyanobacteria differentiate a second cell type called akinete (Adams and Duggan, 1999). Akinetes are cells that help the organism to survive under conditions inappropriate for growth. They are especially resistant against desiccation.

Heterocystous cyanobacteria are mostly known from freshwater and brackish environments, while non-heterocystous cyanobacteria are the dominant diazotrophs in the tropical and sub-tropical ocean (Stal, 2009). Non-heterocystous diazotrophic cyanobacteria are found globally in benthic microbial ecosystems such as microbial mats, although heterocystous cyanobacteria occur in coastal microbial mats that are frequently exposed to low salinity (Severin et al., 2010). This is the case in microbial mats that develop in the higher reaches of the intertidal area where upwelling groundwater and rain are more important than occasional immersion by seawater. Estuarine intertidal sediments are special because the salinity of the water depends on how far upstream they are located. In some estuaries, tidal inundation is only by freshwater, in others by brackish water at various salinity levels.

We have therefore investigated an estuarine microbial mat in the Lagoa dos Patos estuary (Brazil) that is dominated by heterocystous cyanobacteria. This estuary is the biggest lagoon in Brazil and the largest barrier-lagoon of South America (about 10,360 km^2^) (Möller and Castaing, 1999). The climate is temperate, salinity varies between almost 0 and 34 and is mainly influenced by river discharge and wind regime, which control the tidal level and entry of salt water. We isolated from this mat a heterocystous cyanobacterium with unusual properties. Here we report on the isolation and characterization of this benthic cyanobacterium, which may represent a new genus that is closely related to the toxic planktonic *Cylindrospermopsis raciborskii*.

## Materials and Methods

### Strain Isolation and Identification

**FIGURE 1:**
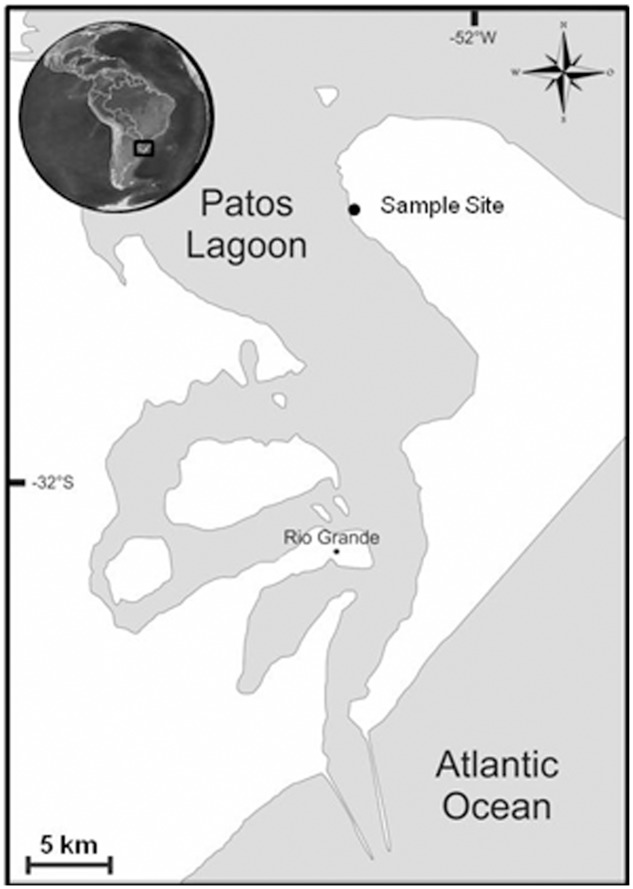
Map of the Patos Lagoon and the location of the sample site.

The cyanobacterium was isolated in 2012 from intertidal sediment in Lagoa dos Patos (31°52.711′S; 52°3.917′W; RS – Brazil) (**Figure 1**). The sediment was sampled and used to inoculate Petri dishes with BG11 medium (Rippka et al., 1979) solidified with 2% agarose (Andersen and Kawachi, 2005). The colonies that appeared were inoculated in liquid BG11 medium and the strain was isolated by picking single trichomes using a micropipette (Andersen and Kawachi, 2005). The strain was kept at the culture collection of cyanobacteria and phycotoxins laboratory (LCF) of the Institute of Oceanography (IO) of the Federal University of Rio Grande (FURG), Brazil. The strain was cultured in cell tissue culturing flasks with BG-11 medium at 25°C, 25 μmol m^-2^ s^-1^ of photon flux density, and an alternating light-dark cycle of 16:8 h. Before the start of the experiments, the strain was acclimated for three months in BG11^0^ (lacking nitrate) in order to induce nitrogenase activity and allow for diazotrophic growth, while keeping temperature and light conditions unaltered. Every 24 h the cultures were homogenized by shaking the flasks. The uni-cyanobacterial (but not axenic) strain was identified as a filamentous heterocystous cyanobacterium and assigned the collection number RS0112. The identification was done by light microscopy with the aid of the taxonomic literature. Sequencing the 16S rRNA gene supported the identification of the isolate.

### Scanning Electron Microscopy

The culture was filtered through 25 mm Nucleopore membrane filters (Whatman Inc., New Jersey, United States) followed by dehydration through a series of alcohol (25%, 50%, 75%, 95%, 100% v/v). The samples were viewed under an SEM Jeol JSM 6610LV, coated with gold at 20 kV at the CEME-SUL of the Federal University of Rio Grande (FURG – Brazil).

### Clone Libraries, Sequencing, and Sequence Analysis

The identification of strain RS0112 was based on its 16S rRNA gene sequence. The DNA of the strain was isolated from the cells obtained from centrifuging 10 ml of culture by using the PowerSoil DNA Isolation Kit (MO-BIO Laboratories Inc., Carlsbad, CA, United States) following the instructions of the manufacturer. The fragment size and yield of the extracted DNA were verified by gel electrophoresis on a 2% agarose gel as well as by measuring the absorption at 230, 260, and 280 nm on a NanoDrop ND 1000 (NanoDrop Technologies Inc., Wilmington, DE, United States) spectrophotometer.

The 16S rRNA gene was amplified using primers 8F (5′-AGA GTT TGATCM TGG CTC AG-3′)/1492R (5′-GGT TAC CTT GTT ACG ACT T-3′) (Weisburg et al., 1991). Thermal conditions for PCR were 15 min at 95°C followed by 35 cycles of 1 min at 95°C, 30 s at 55°C, and 1.5 min at 72°C, followed by an extension period of 7 min at 72°C. The 16S rRNA gene PCR products were purified with DNA Clean and Concentrator (Zymo Research, United States).

A clone library of the 16S rRNA gene was constructed using TOPO-TA pCR kit (Life Technologies, Thermo Fisher Scientific, Carlsbad, USA) according to the manufacturer’s instructions. *Escherichia coli* (TOP10F’) transformants were plated on LB-IPTG-Xgal Medium with ampicillin, incubated overnight at 37°C, followed by 4 h at 4°C in order to improve the visibility of the blue colonies of non-transformed cells. Ten white colonies were picked and the plasmid DNA was extracted (GeneJET Plasmid Miniprep kit, Life Technologies, Thermo Fisher Scientific) and checked by PCR for containing the insert using M13 primers and subsequently sequenced.

A contiguous 1,485 bp sequence of the 16S rRNA gene was obtained. BLAST analysis using megablast (*e*-value 1e^-10^, word size 32, maximum matches in the query range 1, gap costs linear, and filter of low complexity regions) and the “16S rRNA (Bacteria and Archaea)” NCBI database indicated 96% identity with *Anabaena cylindrica* strain PCC7122. The affiliation of RS0112 to heterocystous cyanobacteria was further explored by 16S rRNA gene phylogenetic analysis. GenBank accession number is KY038574.

Representative sequences of 10 heterocystous genera (*Anabaena*, *Aphanizomenon*, *Calothrix*, *Cylindrospermopsis*, *Fischerella*, *Mastigocladus*, *Nodularia*, *Nostoc*, *Scytonema*, and *Stigonema*), 1 filamentous non-heterocystous genus (*Pseudanabaena*) and 1 unicellular (*Gloeobacter*) genus of cyanobacteria were used for inferring the evolutionary relationship. The out-group sequence used for rooting the phylogenetic tree belongs to *Bacillus* sp. SS9. The raw ClustalW alignment (Supplementary Material) included sites with missing/ambiguous data and gaps. 665 overlapping sites out of a total of 2,019 sites were considered for the phylogenetic model (Thompson et al., 1994). The phylogenetic analysis was conducted in MEGA7 (Kumar et al., 2016) using the UPGMA method with bootstrap test of 100 replicates. The evolutionary distances were based on the number of base differences per sequence. The position containing gaps were discarded. The final dataset contained 665 positions.

### Salinity Treatment

This experiment was designed to study growth and dinitrogen fixation of strain RS0112 under three different salinities that are relevant for the natural intertidal environment in which this cyanobacterium is thriving. Freshwater (salinity ∼0) conditions were obtained by using medium BG11^0^, artificial seawater medium (salinity ∼30) was ASN3^0^, and the intermediate (‘brackish’) medium, BSN^0^ was a 1:1 mixture of these media (salinity ∼15). The ‘^0^’ denotes that the media were devoid of combined nitrogen, except for a low amount that comes with ferric ammonium citrate, which is a component of the media. The composition of the media can be found in Rippka et al. (1979). The stock culture of RS0112 was maintained in medium BG11^0^. The experimental cultures (500 ml) were inoculated with 5% of stock culture. All cultures were grown in triplicate. The cultures were grown in disposable sterile cell tissue culture flasks and incubated in a DBO incubator (JumodTrom 304) at 25^o^C and photon flux density of 25 μmol m^-2^ s^-1^ and a 16 h photoperiod and 8h of darkness.

**FIGURE 2:**
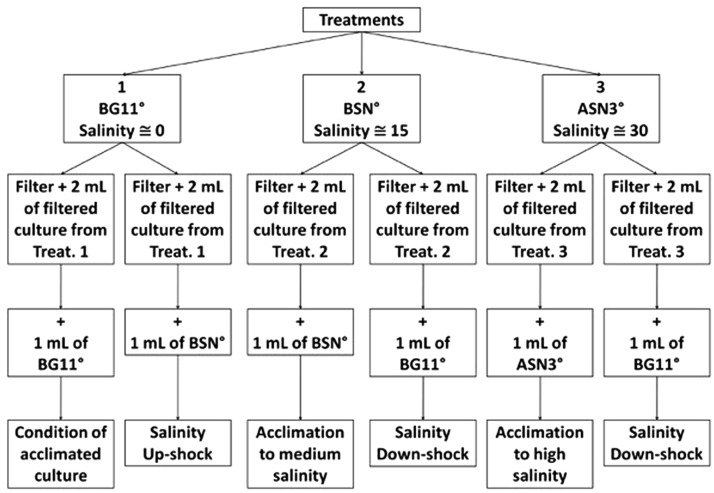
Experimental design for the incubation and culturing of strain RS0112 under three levels of salinity.

At intervals of 48 h, at 2 h after the start of the light period, 8 ml culture was sampled from each flask, which were subsequently divided in four subsamples of 2 ml. One sample was fixed with 0.5% Lugol and kept in the dark for cell counting. The other three samples were filtered on 25 mm GF/F filters. The filtrates were pooled and frozen at –20°C for analysis of dissolved nitrogen (ammonium, nitrate, and nitrite). Two filters were used for the acetylene reduction assay (ARA) to measure nitrogenase activity. The filters were placed in 10-ml borosilicate glass vials and 0.5 ml of culture medium was added. One filter obtained the same medium where it was grown in while the other filter obtained a different medium as to expose it to a different salinity according the scheme in **Figure 2**. Freshwater medium was exposed to brackish salinity, brackish medium was exposed to freshwater, and seawater medium was exposed to freshwater. After the ARA, the chlorophyll *a* content was measured.

### Nitrogenase Activity Assay

Nitrogenase activity was measured by the ARA (Stewart et al., 1967). Two different approaches were tested, namely the filter- and the cell suspension method (Staal et al., 2001). The linearity of the ARA with regard to biomass (chlorophyll *a*-content) was checked and confirmed. Different volumes of culture (0.5, 1.0, 2.0, 4.0, 6.0 ml) were filtered on glass microfiber filters (25 mm, Whatman GF/F) and transferred to the assay vial (10-ml Chrompack) containing 0.5 ml of BG11^0^ and sealed with a butyl rubber stopper. In the second approach the culture was aseptically centrifuged and re-suspended in sterile BG11^0^ in order to obtain similar concentrations of chlorophyll per 2 ml suspension compared to the filtered cultures. Two ml of these re-suspended cultures were directly added into the assay vial and sealed. After the assay, the filters and suspensions were collected for the actual chlorophyll *a* content (see below).

The ARA was started by injecting 1.5 ml of acetylene gas in the vial, using a disposable 2-ml syringe and the vials were incubated for 1 h in the incubator where the cultures were growing and hence exposed to the same conditions of light and temperature (25°C, 25 μmol m^-2^ s^-1^).

Ethylene and acetylene were measured gas chromatographically using a Compact GC^TM^ (Global Analyser Solutions, The Netherlands), equipped with a flame ionization detector (FID). The pressure and the split flow were 125 kPa and 5 ml min^-1^, respectively. The flows of hydrogen and air for the FID are 30 and 300 ml min^-1^, respectively. The oven temperature was set at 50°C; the valve (injector) and FID temperatures were 80 and 110°C, respectively. The gas chromatograph was equipped with a 50-μl sample loop that was flushed with 1 ml of gas sample from the incubation vial. Under these conditions, the retention times for ethylene and acetylene were 95 and 135 s, respectively. The gas chromatograph was calibrated with custom-made standard mixtures of ethylene and acetylene. The contamination of acetylene with ethylene was determined and all measurements were corrected for it. Acetylene was used as an internal standard according to Stal (1988).

### Cell Counting

Cells were counted in a Sedgwick-Rafter chamber using an inverted light microscope at 40× magnification (AxioVert A1, Zeiss, Germany). Vegetative cells, heterocysts, and akinetes were counted in the trichomes and expressed as cells ml^-1^.

### Growth

Growth of the culture was linear as is usually the case with phototrophic microorganisms. This is caused by a - from generation to generation – continuously decreasing rate of exponential growth of the cells caused by light limitation. The growth rate was calculated using the exponential model (lnN_t2_–lnN_t1_)/(t2–t1) from each set of two (t1 and t2) successive measurements (N) of cell counts (division rate) or chlorophyll concentration (growth rate).

### Dissolved Nitrogen

Samples were filtered on Whatman microfiber^®^ filter GF/F and stored frozen at –20°C until analysis for ammonium (NH_4_^+^), nitrate (NO_3_^-^), and nitrite (NO_2_^-^). Analysis was done using a SEAL QuAAtro segmented flow analyzer according to the procedures provided by the manufacturer (Jodo et al., 1992; Aminot et al., 2009).

### Total Organic Carbon and Total Nitrogen

Pre-weighted GF/F filters (Whatman) (dried 24 h at 60°C) were used to sample and to filtrate 2 ml of the cultures at day 30 and subsequently dried and weighted again. Dry weight could only be measured in the BG 11 medium because the salt from the saline media contributed too much to the dry weight. The filters were subsequently freeze-dried, ground to powder, and analyzed for total carbon and total nitrogen using an organic element analyzer (Interscience Flash 2000) (Nieuwenhuize et al., 1994).

### Measurement of Chlorophyll *a*

Chlorophyll *a* was extracted from the ARA assays as follows. The biomass was filtered on a 25 mm GF/F filter and incubated with 0.5 ml culture medium. After the ARA, the culture medium was centrifuged in an Eppendorf tube at 10,000 rpm for 5 min and the pellet suspended in 96% ethanol and added to the filter. The chlorophyll was extracted in 6 ml ethanol for 24 h at 4°C in the dark. The extract was centrifuged again and the supernatant measured spectrophotometrically at 665 nm (Ritchie, 2006). The chlorophyll concentration was transformed to μg Chl-*a* ml^-1^ (culture).

## Results

**FIGURE 3:**
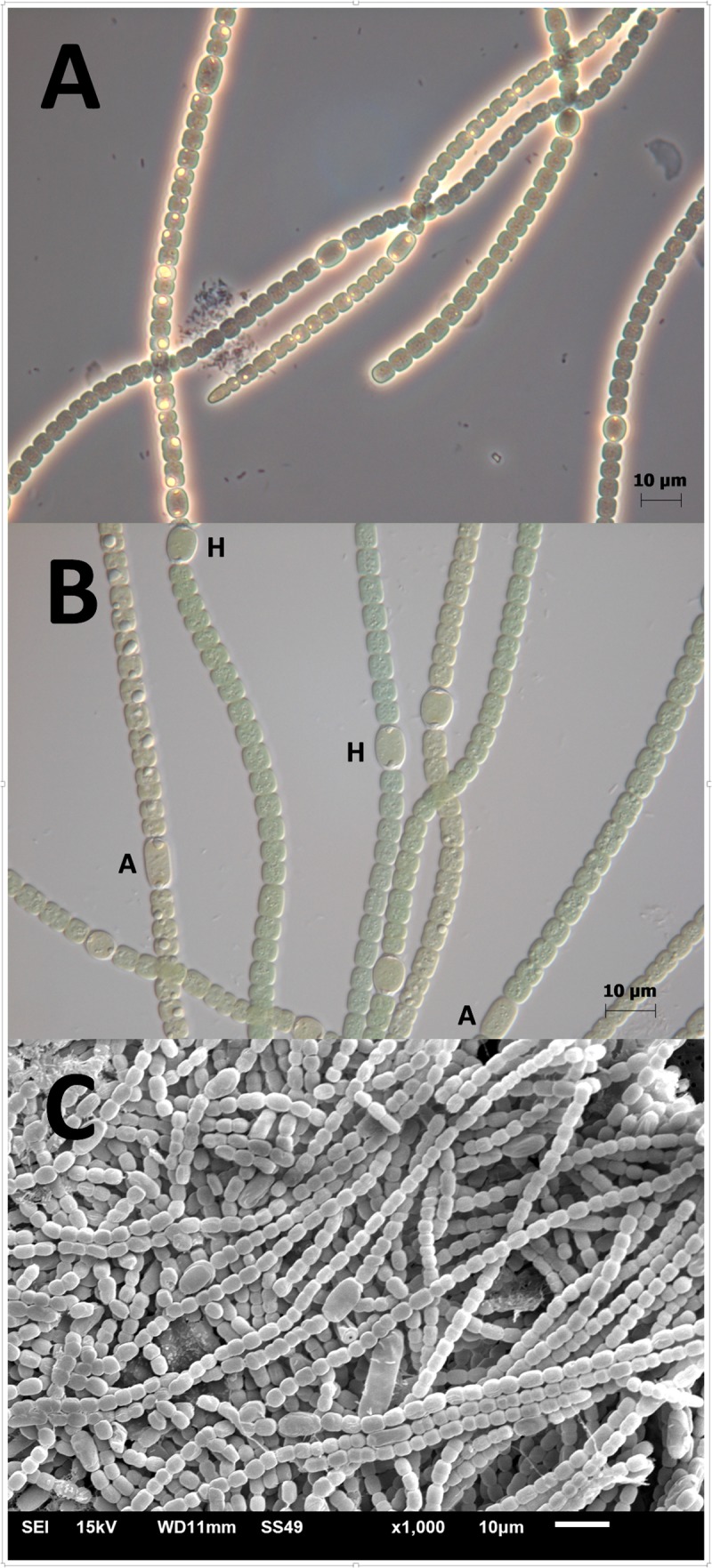
Strain RS0112. **(A)** Magnification 40× phase contrast; **(B)** magnification 63× differential interference contrast. H = heterocyst; A = akinete. The inclusions are most likely cyanophycin granules. **(C)** Scanning Electron Microscopy image.

**FIGURE 4:**
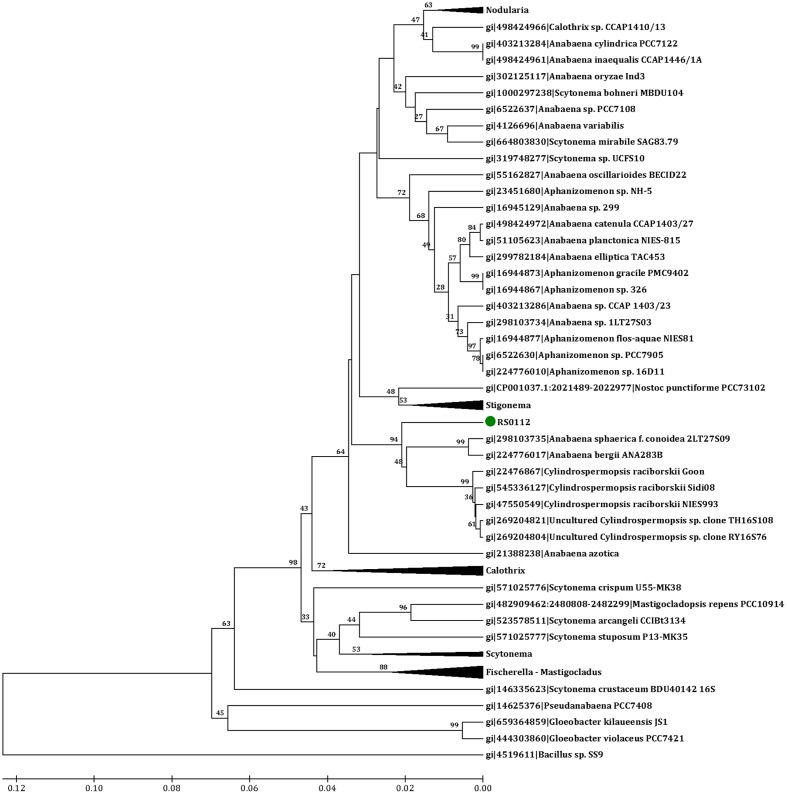
Phylogenetic tree of cyanobacterial 16S rRNA gene sequences used for inferring the affiliation of strain RS0112 to heterocystous cyanobacteria. Analysis based on the alignment of 64 heterocystous, 1 filamentous, and 2 unicellular cyanobacteria and 1 non-cyanobacterial sequences. Evolutionary distance estimated using the number of differences and UPGMA method with bootstrap 100. Genes IDs and bootstrap values are indicated.

The isolated cyanobacterium was filamentous, differentiated heterocysts and akinetes, and grew diazotrophically (at the expense of dinitrogen as the only source of nitrogen) (**Figure 3**). The phylogenetic analysis placed the isolate RS0112 in a cluster with the heterocystous cyanobacterium *Cylindrospermopsis raciborskii* (**Figure 4**).

**FIGURE 5:**
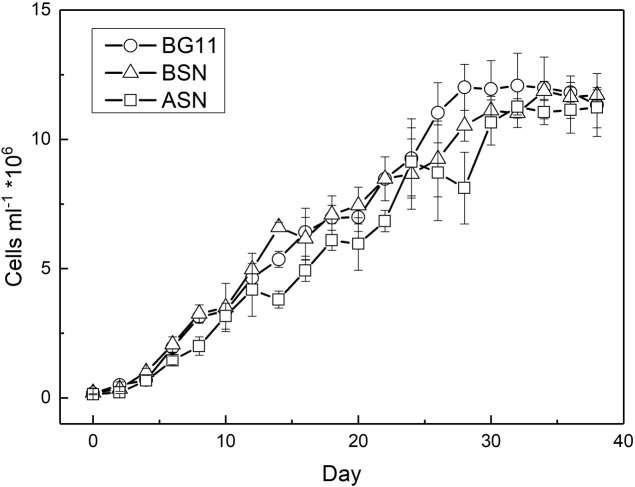
Growth of strain RS0112 measured as cell abundance.

**FIGURE 6:**
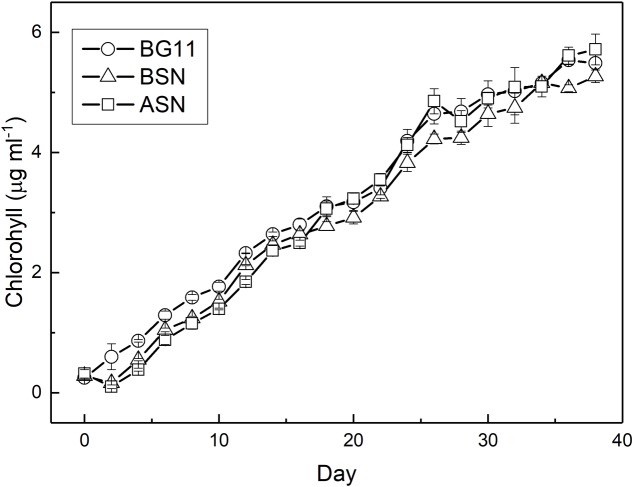
Growth of strain RS0112 measured as chlorophyll content.

RS0112 was grown at three salinities, ∼0, ∼15, and ∼30 (freshwater (BG11^0^), BSN^0^ (1:1 BG11^0^:ASN3^0^), and at full salinity seawater (ASN3^0^), respectively) without combined nitrogen and, hence, depending on the fixation of atmospheric dinitrogen (N_2_). As indicated by the time-changes in abundance (**Figure 5**), all cultures observed an early short phase of exponential growth that progressively tapered. When reaching the stationary phase, the cultures reached an abundance of ∼10^7^ cells/ml. In terms of cell abundance there were no differences observed between the three tested salinities. Growth measured as the increase of chlorophyll *a* was similar at any of the three tested salinities and was linear rather than exponential (**Figure 6**). The chlorophyll content per cell was high at the start of the experiment at all three salinities (2.3 ± 0.2, 2.8 ± 0.3, and 4.7 ± 1.1 pg/cell in BG11^0^, BSN^0^, and ASN3^0^, respectively). This was due to the inoculum that had accumulated chlorophyll during the stationary phase. The chlorophyll content of the cells rapidly decreased during the first days of the culture and it diluted out to ∼0.5 pg/cell. After that it remained more or less constant until the culture entered the stationary phase around day 30. The average values for BG11^0^ was 0.45 ± 0.05 pg/cell (after 10 days of culturing) and 0.62 ± 0.46 pg/cell (over the whole 38 days); for BSN^0^ this was 0.43 ± 0.05 pg/cell (after 10 days of culturing) and 0.49 ± 0.23 pg/cell (over the whole 38 days); for ASN3^0^ was 0.51 ± 0.08 pg/cell (after 10 days of culturing) and 0.61 ± 0.43 (over the whole 38 days). At the start of the culture no chlorophyll was synthesized. The ASN3^0^ culture seemed to have slightly higher chlorophyll compared to the other two, but this was not significant. Overall the chlorophyll content amounted on average 0.46 pg/cell.

**FIGURE 7:**
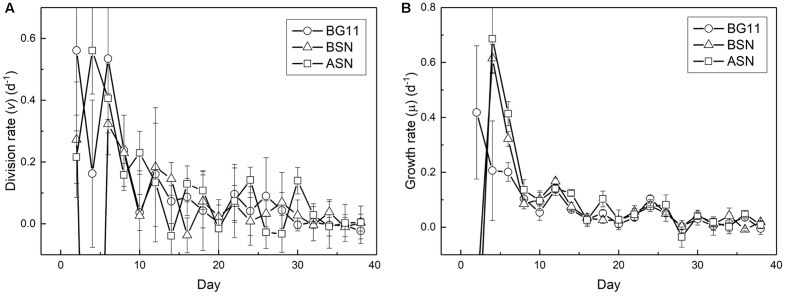
Change in division **(A)** and growth **(B)** rates during culturing strain RS0112.

The fact that the growth curve appears linear is because the ever-decreasing growth and division rate, probably caused by light limitation (**Figure 7**). The highest growth and division rates were observed immediately after inoculating the culture, after which the division rate decreased to zero at day 30. The chlorophyll content of the cultures was still increasing in the stationary phase. The maximum growth rate (μ_max_) measured was ∼0.6 d^-1^. The average growth rates over the whole 38 days were 0.08, 0.05, and 0.05 d^-1^ for the cultures grown in BG11^0^, BSN^0^, and ASN3^0^ medium, respectively and the average division rates were 0.11, 0.05, and 0.12 d^-1^ for BG11^0^, BSN^0^, and ASN3^0^, respectively.

The cellular carbon and nitrogen content was measured at day 32. The carbon and nitrogen content did not differ significantly between the three growth-media and amounted 17 ± 1.0, 19 ± 1.1, and 19 ± 0.8 pg C cell^-1^ and 2.9 ± 0.1, 2.9 ± 0.2, and 2.8 ± 0.1 pg N cell^-1^ in BG11^0^, BSN^0^, and ASN3^0^, respectively. The average cell diameter was ∼5 μm and assuming a sphere, the average cell volume was ∼65 μm^3^. The C and N concentration was on average 282 fg C μm^3^ and 44 fg N μm^3^. The C:N ratios increased slightly with salinity of the growth media from 5.9, 6.3, and 6.6 in BG11^0^, BSN^0^, and ASN3^0^, respectively and were close to Redfield (Redfield, 1958) and slightly below the 7.7 reported by Geider and LaRoche (2002). Hence, the cultures were certainly not depleted by nitrogen.

**FIGURE 8:**
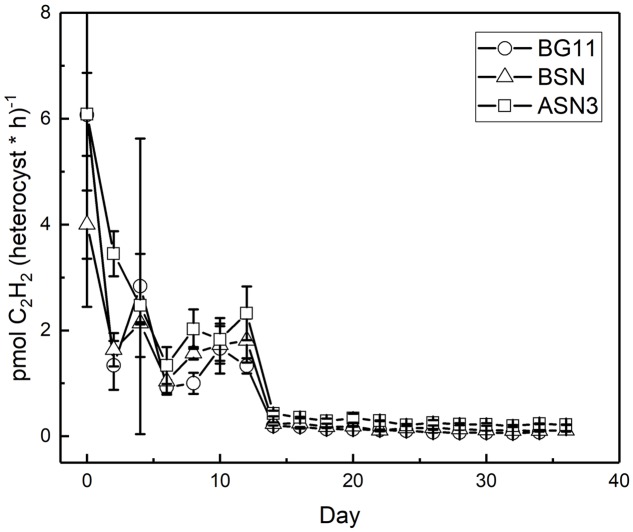
Nitrogenase activity per heterocyst during culturing of strain RS0112 at three different salinities.

**FIGURE 9:**
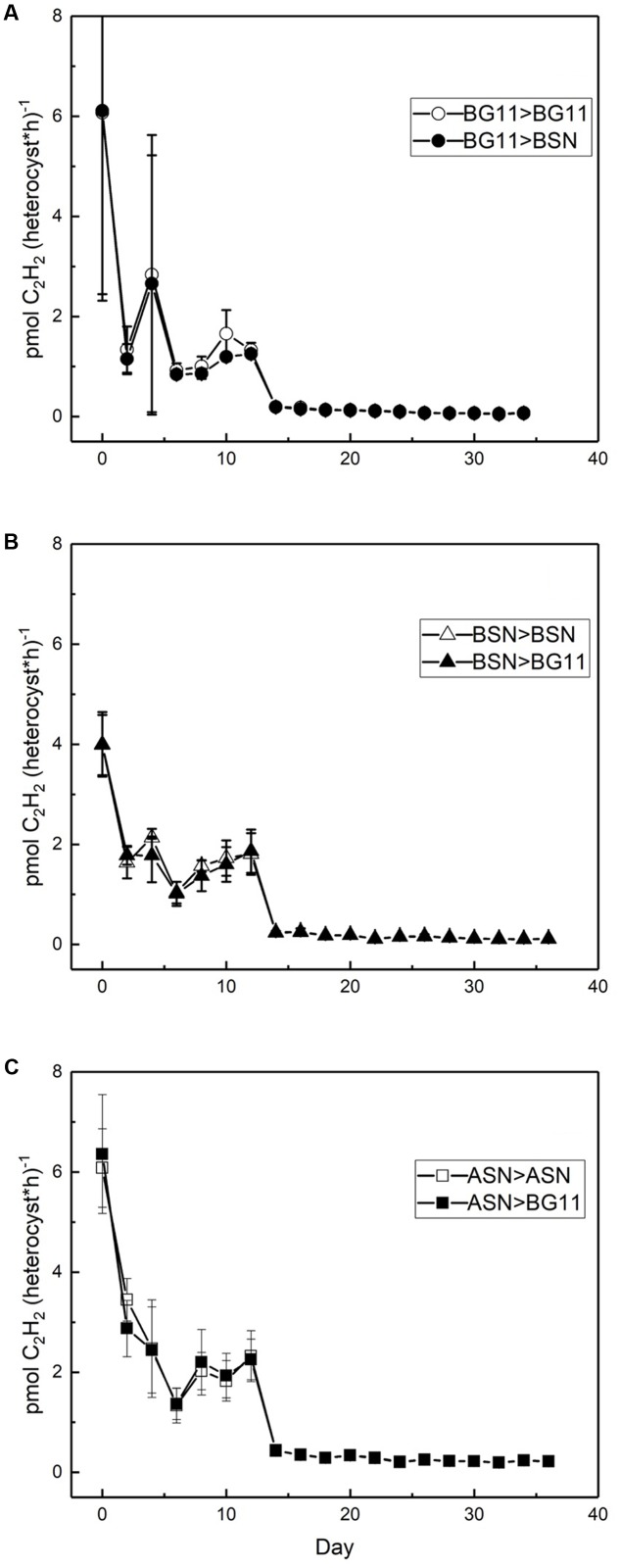
Effect of a salinity down-shock on nitrogenase activity per heterocyst during culturing of strain RS0112 at three different salinities. **(A)** BG11: 0‰, **(B)** BSN: 15‰, **(C)** ASN: 30‰.

Nitrogenase activity was measured using the ARA. There was a linear correlation between chlorophyll content of the culture and nitrogenase activity up to 18 and 28 μg chlorophyll in the filter and suspension incubations, respectively (see Materials and Methods). The filter and suspension incubations did not give a significant difference and the activity was determined as 0.4 μmol C_2_H_2_ (mg Chl)^-1^ h^-1^. Nitrogenase activity was measured during growth at three salinities and expressed per heterocyst (**Figure 8**). High nitrogenase activities were only observed during the initial phase of growth and were highest in full salinity seawater (6.0 ± 0.8 pmol C_2_H_2_ (heterocyst^∗^h)^-1^, intermediate in the brackish medium (4.0 ± 0.8 pmol C_2_H_2_ (heterocyst^∗^h)^-1^ and lowest in freshwater medium (6.1 ± 3.6 pmol C_2_H_2_ (heterocyst^∗^h)^-1^ (in the latter case the high standard deviation was caused by unreliable heterocyst counting at low abundance). After the initial peak, nitrogenase activity dropped to 3, 2, and 1 pmol C_2_H_2_ (heterocyst^∗^h)^-1^ in BG11^0^, BSN^0^, and ASN3^0^, respectively, from day 2, and to 0.2, 0.1, and 0.07 pmol C_2_H_2_ (heterocyst^∗^h)^-1^ in BG11^0^, BSN^0^, and ASN3^0^, respectively, from day 14, and stayed at these low levels. Salinity shocks hardly affected nitrogenase activity in RS0112 (**Figure 9**).

**FIGURE 10:**
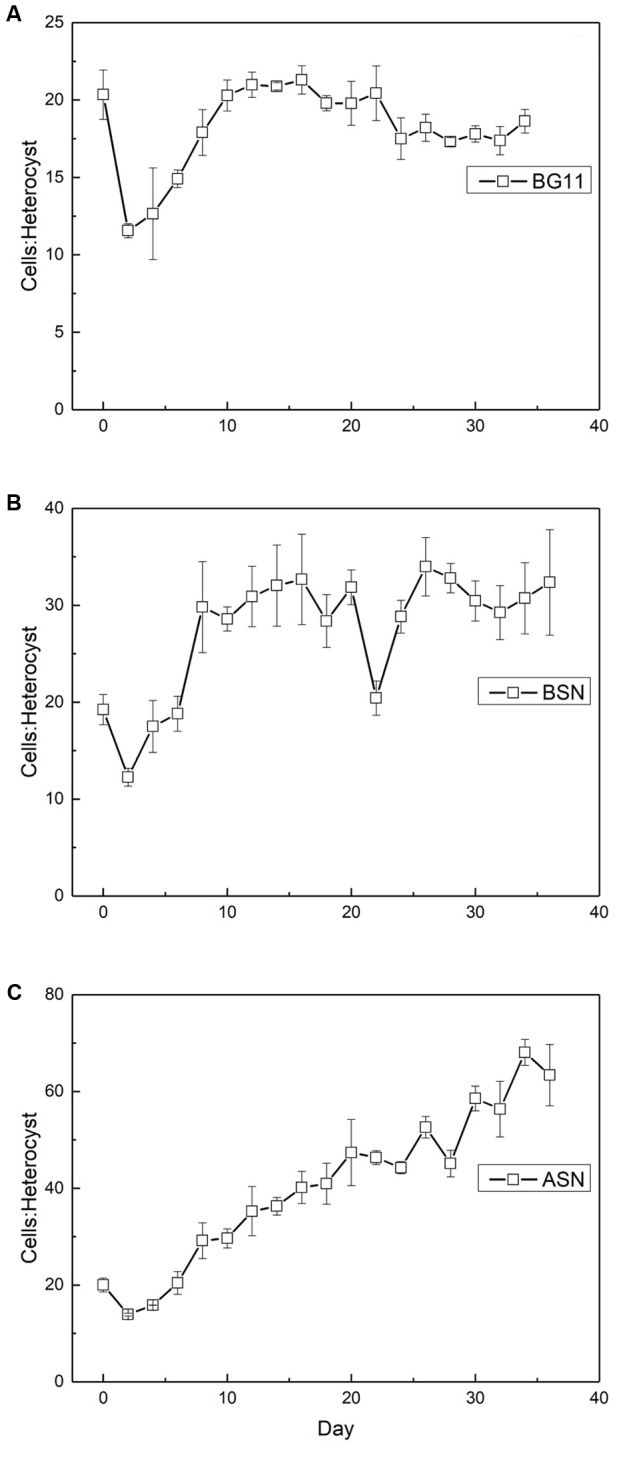
The ratio of vegetative cells to heterocysts in strain RS0112 cultured under three different salinities. **(A)** BG11: 0‰, **(B)** BSN: 15‰, **(C)** ASN: 30‰.

The ratio of vegetative cells per heterocyst showed interesting differences. At the start of the culture the ratio was about 20 vegetative cells per heterocyst. At all three salinities, this number initially decreased to ∼12, indicating an increase in the number of heterocysts and coinciding with the peak in nitrogenase activity. In freshwater medium, this number subsequently increased back to ∼20 cells per heterocyst. In the brackish medium, the number of vegetative cells per heterocyst increased to ∼30 (meaning fewer heterocysts) and in full salinity seawater as many as 60 vegetative cells per heterocyst were counted (**Figure 10**).

**FIGURE 11:**
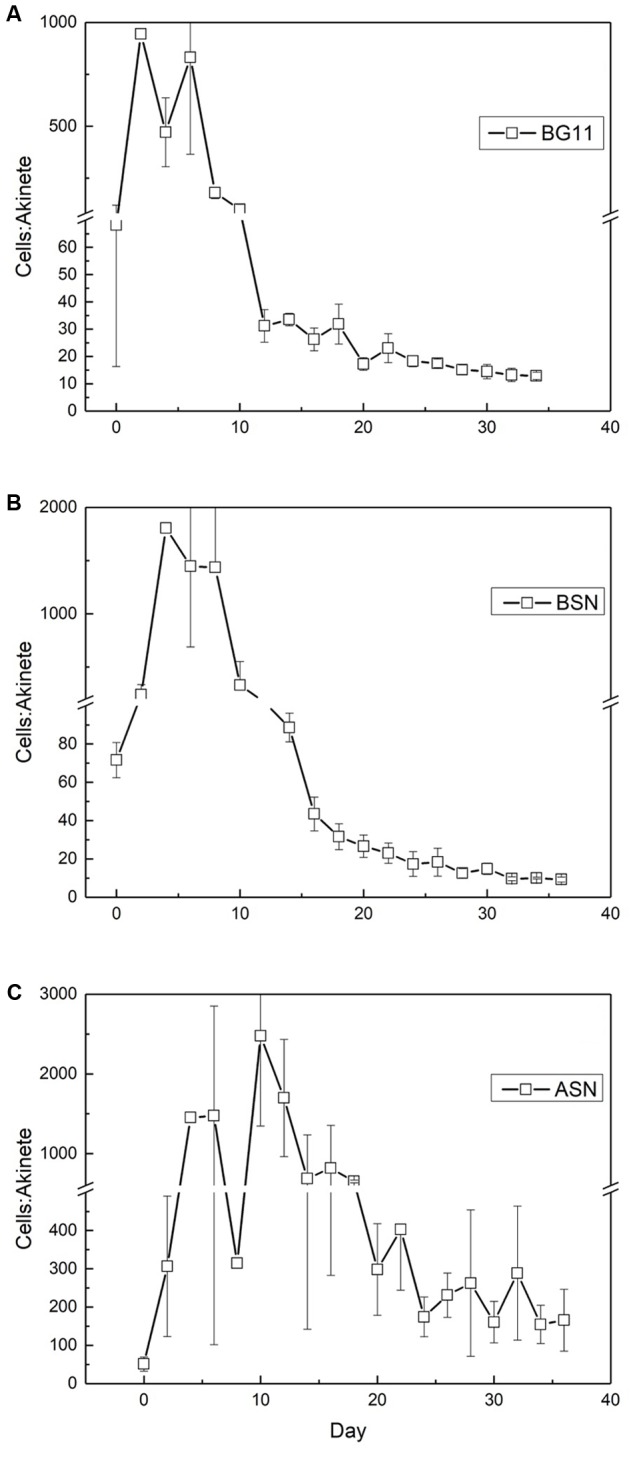
The ratio of vegetative cells and akinetes in strain RS0112 cultured at three different salinities. **(A)** BG11: 0‰, **(B)** BSN: 15‰, **(C)** ASN: 30‰.

Similar as was the case with the number of heterocysts, the number of akinetes also showed an interesting trend, which differed between the three salinities. The starter culture contained 50–70 vegetative cells per akinete, which increased in all cultures, concomitant with the peak in nitrogenase activity (**Figure 11**). The maximum number of vegetative cells per akinete was different between the three cultures. In freshwater about 1000 vegetative cells were counted per akinete, while in brackish water and full-salinity seawater this number doubled. This means that under higher salt conditions the culture formed fewer akinetes. In the later stages of growth, the number of vegetative cells per akinete decreased and eventually reached 10–15 in freshwater and brackish medium but remained 10-fold higher in full-salinity seawater medium.

**FIGURE 12:**
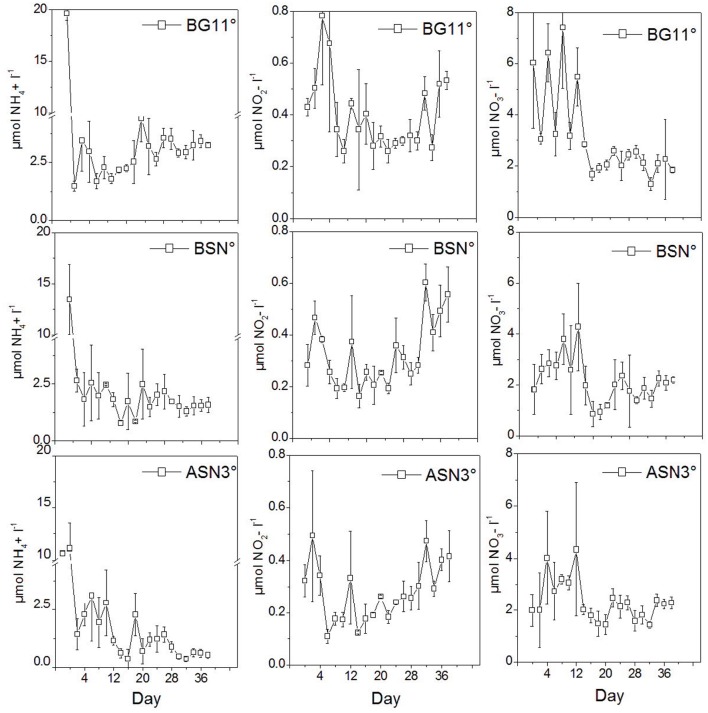
The concentrations of NH_4_^+^, NO_2_^-^, and NO_3_^-^ in the media during culturing strain RS0112.

The concentrations of ammonium, nitrite and nitrate in the media during growth were low (**Figure 12**). Ammonium was 10–20 μM upon inoculation, which is due to the iron ammonium citrate in the medium (6, 4.5, and 3 mg l^-1^ in BG11^0^, BSN^0^, and ASN3^0^, respectively). This ammonium is almost completely depleted in ASN3^0^ at the end of the culturing (<0.5 μM), a little less in BSN^0^ (∼1.5 μM), while after an initial decrease in BG11^0^, the concentration of NH_4_^+^ increased to ∼3 μM. The concentration of nitrate was ∼2 μM upon inoculation in BSN^0^ and ASN3^0^ and ∼6 μM in BG11^0^, increased slightly during the first 12 days of culturing but then again decreased to the value of ∼2 μM. Nitrite was well below 0.5 μM. It decreased slightly during the first week of culturing but then increased slightly during the subsequent culturing. These data show that the growth media were virtually depleted in combined nitrogen.

## Discussion

Strain RS0112 seems to be phylogenetically related to *C. raciborskii*, a heterocystous cyanobacterium that often forms toxic blooms in freshwater lakes (Stucken et al., 2009) (**Figure 4**). Although *C. raciborskii* was originally confined to tropical and subtropical lakes, it has more recently also been found in temperate lakes (Mehnert et al., 2014). This extension of its distribution has been attributed to climate change. *C. raciborskii* contains gas vesicles, which are typical for aquatic filamentous or colony-forming unicellular cyanobacteria. RS0112 is a benthic cyanobacterium and does not contain gas vesicles and may be a new genus related to *Cylindrospermopsis* (**Figure 4**). A similar case is known for the heterocystous cyanobacterium *Nodularia spumigena*, which has a toxic planktonic variety with gas vesicles and a non-toxic benthic type that lacks gas vesicles (Lyra et al., 2005).

Batch cultures of RS0112 grew exponentially during the first week and then continued at slower pace for approximately 4 weeks after which the culture entered the stationary phase (**Figure 5**). The growth measured as the increase in chlorophyll *a* was linear throughout culturing. The cellular chlorophyll content is not constant and increases when self-shading limits light harvesting (Xu and Juneau, 2016). Hence, during exponential growth the cellular content lags behind the cell division, while during the stationary phase chlorophyll content still increases. Therefore, chlorophyll *a* is not the best parameter to measure growth in cyanobacteria because it is not a great measure of biomass. RS0112 grew well in freshwater and full seawater salinity medium, although slightly less in the latter. Most heterocystous cyanobacteria are freshwater organisms and even those isolated from marine sources usually grow well in freshwater medium (Stal, 2009). The cellular chlorophyll content was not affected by the salinity of the medium, which indicated that cultures grown in full salinity seawater medium accumulated relatively more chlorophyll. This could hint to cells compensating the slightly lower growth in full salinity medium with higher chlorophyll and consequently higher light harvesting capacity.

While the growth rate was high during the first week of culturing it decreased thereafter until it became zero (in the stationary phase). The so-called linear growth phase that is typical for phototrophic microorganisms is caused by light limitation (Westwood and Ganf, 2004). Growth by binary cell division is by definition exponential. The growth rate decreases steadily because self-shading continuously decreases light harvesting, which is not compensated by the increase of chlorophyll *a*.

Nitrogenase activity was highest during the exponential phase of growth, but although growth was slightly lower in full salinity seawater medium, nitrogenase activity in the heterocysts was higher. Clearly, nitrogenase activity increased with salinity and was lowest in freshwater medium (in the latter the high standard deviation makes the day 0 data point unreliable). This is unusual because heterocystous cyanobacteria are in general freshwater or brackish organisms. However, a higher nitrogenase activity can be explained by the lower dissolved oxygen concentration in saline medium. Nevertheless, this did not lead to higher growth, probably because another factor than nitrogen limited growth. Once the culture entered the linear growth phase, nitrogenase activity per heterocyst was low but still somewhat higher at the higher salinities. Salinity shocks did not affect nitrogenase activity in the heterocysts, confirming that this strain is adapted to full seawater salinity.

The frequency of heterocysts was highest in freshwater medium and decreased with salinity. The heterocyst frequency was around 5% during the exponential growth phase in the three different salinity media, which is what is normally found in heterocystous cyanobacteria (Brown and Rutenberg, 2012). Immediately after inoculation the heterocyst frequency increased to ∼8%, coinciding the high nitrogenase activity and the high demand for nitrogen during the period of exponential growth. However, this frequency subsequently decreased in freshwater medium to ∼5%, to ∼3% in brackish medium, and to ∼0.7% in full salinity seawater. Obviously, the nitrogen demand could be covered by dinitrogen fixation in fewer heterocysts when the salinity is higher. This agrees with the higher nitrogenase activity per heterocyst at higher salinities. This may be related to the lower dissolved oxygen concentration in saline water, requiring less reducing equivalents for scavenging oxygen that is necessary to prevent the inactivation of nitrogenase (Stal, 2009).

Strain RS0112 produced akinetes. Akinetes are spore-like cells that serve the survival of the organism under conditions that are not favorable for growth. Low light intensity has been identified most frequently as the main factor that triggers akinete formation, although also temperature changes and phosphate limitation has been reported as important factors (Moore et al., 2005; Perez et al., 2016). Akinetes are not heat resistant as is the case with bacterial spores, but withstand long periods of desiccation. Akinetes are also resistant against UV irradiation. They accumulate cyanophycin (multi-L-arginyl-poly-[L-aspartic acid]), glycogen, and or neutral lipids in a strain specific manner. When conditions allow, akinetes germinate, and produce short motile hormogonia that migrate away and subsequently develop mature trichomes (Adams and Duggan, 1999). Akinetes may develop adjacent to heterocysts or halfway between two heterocysts, depending on the species. In the absence of heterocysts, akinetes form randomly and eventually all cells may develop into an akinete, although under some conditions less than 100% of the cells form an akinete. As expected, akinetes were rare during the exponential growth phase in RS0112, amounting ∼1‰ in freshwater medium and even less in the saline media. During the linear growth phase the number of akinetes increased but then remained constant during the stationary phase at ∼10%. In full salinity seawater medium only ∼0.5–1% of the cells developed an akinete. Obviously, seawater repressed the formation of akinetes under otherwise equal conditions, similarly as is the case with the heterocyst. It has been suggested that the heterocyst evolved from the akinete and that the differentiation of both cell types is under similar control (Adams and Duggan, 1999). As far as we know, no systematic research has been published about the effect of salinity on heterocyst and akinete differentiation. While the need to differentiate heterocysts for dinitrogen fixation in full salinity seawater may be less because of the lower solubility of oxygen (Stal, 2009), this argument would not hold for akinetes, unless their differentiation is under the same control.

The C:N ratio showed that the cells were nitrogen replete or even enriched in nitrogen at the stationary phase while the growth media were depleted in ammonium, nitrate and nitrite. The cells contained inclusions that are most likely cyanophycin (**Figures 3A,B**), which also hinted to nitrogen repletion of the cells (Picossi et al., 2004).

## Conclusion

The growth of the heterocystous cyanobacterium strain RS0112 was hardly affected by salinity in the range of freshwater to full salinity seawater although nitrogenase activity per heterocyst was higher at higher salinity. The heterocyst and akinete frequency were considerably lower at higher salinity and this might be related to the lower solubility of oxygen in seawater relative to freshwater. Hence, strain RS0112 seems to be well adapted to growth in an estuarine benthic environment where it is exposed to fluctuating salinity imposed by the tidal currents.

## Author Contributions

PG isolated the strain, designed the experiments, analyzed, and interpreted the results, and wrote parts of the manuscript. JY interpreted the results and wrote parts of the manuscript. MC did the phylogenetic analysis, interpreted the results, and wrote parts of the manuscript. LS designed the experiments, interpreted the results, and wrote the manuscript.

## Conflict of Interest Statement

The authors declare that the research was conducted in the absence of any commercial or financial relationships that could be construed as a potential conflict of interest.
